# Reimagining prenatal physical activity and exercise as potential therapeutic strategy in promoting maternal health

**DOI:** 10.12669/pjms.42.6.12771

**Published:** 2026-06

**Authors:** Daniel Ter Goon, Uchenna Benedine Okafor

**Affiliations:** 1 Daniel Ter Goon, Faculty of Health Sciences, University of Limpopo, Sovenga, South Africa; 2 Uchenna Benedine Okafor, Centre for Health Promotion and Disease Prevention, Edson College of Nursing and Health Innovation, Arizona State University, Phoenix, AZ, USA

**Keywords:** Benefits, Interventions, Patterns, Prenatal physical activity, Sources of information

## Abstract

Promoting prenatal physical activity (PA) is one of the health intervention strategies for improving mother outcomes. However, pregnant women are rarely or inadequately informed about the benefits of prenatal PA engagement, which has an impact on their degree of prenatal PA and participation in low-intensity PA. Prenatal PA is reportedly low in many countries around the world, and this is due to a variety of multidimensional constraints. Health promotional and advocacy intervention strategies such as community campaigns or engagements, roadshows, flyers at health facilities, and incorporating prenatal PA into antenatal care may help to scale up the promotion of prenatal PA among pregnant and postpartum women in a variety of settings. Physical activity and exercise should be reconceptualized as a therapeutic strategy for reducing maternal mortality, rather than being limited to biomedical factors.

## INTRODUCTION

Physical activity and exercise during pregnancy can help improve mother and foetal health outcomes. Physical activity is defined as all muscular muscle activities that result in energy expenditure, while exercise refers to a planned, structured, and repetitive activity.^1^

The key link between these two concepts is that they serve to maintain overall health and achieve therapeutic health outcomes. Therefore, in this article, these two terms are applied interchangeably to define the activeness of pregnant women during pregnancy. The article discusses how to promote prenatal PA and exercise by examining the available information for pregnant women and how their understanding of the benefits influences their engagement in these activities. The objective is to address the following question: What are the potential interventions that could promote prenatal PA and exercise in an ever-changing environmental landscape? Recognising the critical benefits of being active throughout pregnancy, encouraging prenatal activity is beneficial. Interventions aimed at addressing modifiable factors that constrain prenatal PA and exercise are essential for promoting the health and well-being of both the mother and the foetus. This approach aligns with Sustainable Development Goal 3, which focusses on ensuring healthy well-being for all.

Research indicates that consistent PA and exercise during pregnancy enhances the health outcomes for both pregnant women and their infants in different health conditions. Engaging in prenatal PA and exercise during pregnancy benefits both the mother and the foetus.^2^ Participation in prenatal PA and exercise during pregnancy improves maternal and foetal health, decreases pregnancy-related complications, and results in more favourable maternal health outcomes, as well as enhanced obstetric and neonatal outcomes. This includes a reduction in gestational diabetes mellitus (GDM), high blood pressure, urinary incontinence, third- or fourth-degree perineal tears, and macrosomia.^3^-^7^ Additional benefits encompass a reduced likelihood of low birth weight, small gestational age, or high birth weight, caesarean delivery, and an extended gestational age period.^8^,^9^ It also improves mental health by reducing depression, anxiety, and stress.^10^,^11^

Nevertheless, women are often insufficiently informed about prenatal PA and exercise, leading to their passive or non-participation in such activities.^5^,^12^,^13^ Globally, prenatal physical and exercise levels are reportedly low in both developed and developing countries, which may be attributed to a lack of information or misinformation about benefits and perceived fears about miscarriage, contractions, bleeding, and foetal health.^13^-^23^ Moreover, pregnant women receive inadequate information on prenatal activity during antenatal care from health care providers, resulting in persistent ambiguity regarding the benefits of prenatal PA.^5^,^12^

To effectively promote prenatal PA, it is essential to train healthcare providers, as they might lack understanding of the underlying scientific principles pertaining to the subject, rendering them unable to provide scientific evidence-based education and counselling on prenatal physical activity. Consequently, incorporating prenatal PA and exercise into medical or nursing curricula would equip healthcare providers with the expertise to deliver evidence-based prenatal PA prescriptions, guidance, and counselling to pregnant women during clinical and antenatal consultations.

The cultural reconfiguration of prenatal PA and exercise narratives may serve as a fulcrum for promoting PA and exercise among pregnant women. Participation in PA during pregnancy is often discouraged in African contexts owing to concerns of foetal safety, which are rooted in stereotypical cultural beliefs and misconceptions,^5^,^19^,^24^ which often decrease their PA levels during pregnancy.^25^,^26^ Similar misunderstandings have been observed in other global settings, such as Asia and Latin America, where cultural traditions often associate rest with safety during pregnancy.^27^,^28^ These culturally influenced beliefs often lead to decreased PA among expectant mothers, highlighting the importance of developing globally inclusive and culturally aware strategies that address these misconceptions and encourage safe prenatal exercises.

However, scientific research has demonstrated the need of encouraging prenatal PA involvement during pregnancy. Healthcare providers are positioned to deliver prenatal physical counselling and education to pregnant women to discourage sedentarism during pregnancy. The question remains whether healthcare providers possess the requisite scientific knowledge regarding prenatal activity guidelines and prescriptions to effectively deliver this service in healthcare settings. Prioritising pregnant PA and exercise as a component of maternal healthcare during obstetric and antenatal care includes preparing healthcare workers with scientific understanding of prenatal physical guidelines and recommendations. This would allow them to deliver accurate and scientific information and assistance to pregnant women regarding prenatal PA and exercise as part of maternal health care.

Insufficient prenatal PA poses significant risks to both maternal and fetal health. Given the well-documented evidence supporting the role of PA in improving maternal and neonatal outcomes, there is a critical need for context-specific interventions that address barriers, strengthen enablers, and promote active engagement during pregnancy. These interventions should also ensure effective communication of evidence-based information on the benefits and safety of prenatal PA to support informed decision-making and healthier pregnancies.^29^-^32^ More importantly, prioritising prenatal PA in the antenatal health care program is critical for rethinking prenatal PA as a strategy to improving mother and foetal health. Such strategy will aid in improving the pattern of PA participation among pregnant women. Hence, pregnant women tend to engage in low-to-moderate housework and occupational PA; it is time to redefine prenatal PA as a central strategy for improving maternal health outcomes, moving beyond the narrow scope of traditional biomedical approaches.^24^ Achieving this requires that pregnant women are motivated, inspired, and supported to engage in high-intensity physical exercise in accordance with scientific guidelines and supervision.

Now ‘*Where do Pregnant Women get Prenatal PA and Exercise Information from?’* Throughout pregnancy, women actively pursue information that will equip them for their roles as mothers.^33^ As a result, pregnant women tend to seek out pregnancy-related information from various sources. Clearly, prenatal healthcare providers are in a unique position to deliver formal education on antenatal care to pregnant women within their healthcare settings, which includes guidance on prenatal PA and exercise. Therefore, women need not only information about prenatal exercise^33^ but also precise, factual, and scientifically validated information to support the adoption of PA behaviours during and after pregnancy.

The absence of reliable information regarding PA activity frequently drives women to depend on their own beliefs or other questionable sources for guidance during pregnancy. This situation foreshadows a compromising behaviour of PA restriction at the start of a pregnancy and as it progresses, which is linked to an increase in physical discomforts and pelvic girdle pain experienced during pregnancy.^34^-^38^ Navigating this challenge involves equipping prenatal healthcare providers with scientific knowledge regarding prenatal PA and exercise to enable them deliver credible and appropriate advice and counselling specific to a trimester of a woman. Nonetheless, the insufficient guidance on prenatal PA and exercise provided by healthcare providers leads pregnant women to primarily depend on online resources, and social media for their information, which might be misleading.^39^-^41^ In certain cases, healthcare providers may not possess the necessary knowledge and skills to provide effective prenatal PA advice and counselling.^42^-^44^ Empowering and educating prenatal healthcare providers to offer counselling to pregnant women on the importance engaging in regular PA during pregnancy is crucial.

Expectedly, pregnant women would likely regard information on prenatal PA from prenatal healthcare providers as reliable, rather than relying on what they know, friends, family, magazines, and the Internet. Unfortunately, they may receive inaccurate and contradicting information. Internet-based PA advice is frequently inaccurate and can be deceptive.^45^,^46^ This highlights the need of providing pregnant women with appropriate information about what they should know, do, and imbibe in order to engage in optimal prenatal PA and exercise. However, prenatal PA is seemingly neglected in clinical health settings by physicians and other healthcare professionals due to a lack of training.^47^ There is need to involve exercise physiologists, physiotherapists, and certified fitness professionals to assist in providing relevant information to avoid placing excessive pressure on physicians and midwives.^48^ Understanding pregnant women’s prenatal PA and exercise information, as well as the advice they follow, is crucial to enhance healthcare.^49^

Reimagining prenatal PA and exercise for pregnant women entails recommending them to engage in high-intensity activities for improved health outcomes, rather than only low-intensity ones. Pregnant and postpartum women with no medical complications are encouraged to partake in regular aerobic, muscle-strengthening, and gentle stretching exercises, and women who were previously physically active in strenuous activities are encouraged to continue being active, even after delivery.^50^ Based on the foregoing, healthcare providers are key in echoing this message to women during antenatal session, hence, this antenatal session provide an ideal chance to deliver free prenatal advice and counselling.

## CONCLUSION

Insufficient PA during pregnancy can lead to undesirable health issues for both the mother and their children. Research indicates that maintaining a regular exercise routine during pregnancy can result in better outcomes for both mothers and their babies, including fewer pregnancy complications, improved weight management, and enhanced mental health. To achieve these benefits, policymakers must incorporate prenatal exercise into antenatal care (ANC) guidelines, ensure that healthcare providers are well-informed about exercise recommendations, and support community efforts to address cultural and environmental barriers. Public health campaigns should offer clear, evidence-based information on the safety and benefits of prenatal exercise and encourage women to engage in regular PA. Together, these strategies can promote healthier pregnancies, reduce risks for mothers and infants, and shift prenatal care towards a more holistic, preventive model.
